# New Appliances and Things Medical

**Published:** 1905-12-16

**Authors:** 


### NEW APPLIANCES AND THINGS MEDICAL.

[We shall be glad to receive at our Office, 28 & 29 Southampton Street, Strand, London, W.C., from the manufacturers, specimens of all new preparations and appliances which may be brought out from time to time.]

### ARSENFERRATOSE (SYRUPUS FERRATINI ARSENATE.

(C. F. Boehringer and Soehne. Mannheim Waldhof. London: David Thom, Domeier and Company, Limited, 20 and 21 Harp Lane, E.C.)

Arsenferratose is a preparation of iron and arsenic, as the name implies; but what is peculiar about the preparation and what the name does not imply, at any rate to the average reader, is that in this preparation both the iron and arsenic are present in organic combination. The active principle of arsenferratose is a substance known as arsenic ferratin. This substance consists of ferratin combined with arsenic and contains 0.2 per cent. of arsenic. From experiments in vitro it appears that in the process of digestion only a minute trace of this arsenic is split off, 94.5 per cent. of it remaining fixed to the ferratin. We will not enter here into the question of ferratin except to remind our readers that this substance, according to Professor Schmiedeberg, is identical with the proteid iron compound found in the tissues and is absorbed from the digestive tract as such. It would be still further beyond our purpose to enter here into the vexed question of the relative values of the organic and inorganic preparations of iron. We are not told in the literature, which has been accessible to us, how much arsenic ferratin the preparation contains, but we are informed that the average daily dose is one tablespoonful a quarter of an hour after meals, and that this three times daily amounts to a total dose of about four grains of ferratin and of about one-hundredth part of a grain of arsenic. In the hands of German physicians this preparation has given excellent results as a general tonic. It certainly seems to be free from all objectionable properties whatever, and is in our opinion to be recommended.

### ALLENBURYS FEEDER AND PATENT TEAT.

(Allen and Hanburys, Limited, Bethnal Green, London, E.)

We have received from Messrs. Allen and Hanburys one of their well-known Feeders, together with a new teat which they have designed. This teat has a solid rubber rim which, when applied, so grips the neck of the feeder as to prevent the child from pulling the teat off the bottle, no independent fastening being necessary. The advantage gained by this little improvement is considerable, a fact to which we can testify from practical experience. The teat is certainly the most satisfactory one that we have seen.

